# To the proteome and beyond: advances in single-cell omics profiling for plant systems

**DOI:** 10.1093/plphys/kiab429

**Published:** 2021-09-07

**Authors:** Natalie M Clark, James Mitch Elmore, Justin W Walley

**Affiliations:** Department of Plant Pathology and Microbiology, Iowa State University, Ames, Iowa 50011, USA

## Abstract

Recent advances in single-cell proteomics for animal systems could be adapted for plants to increase our understanding of plant development, response to stimuli, and cell-to-cell signaling.

## Main text

Many biological processes involve the coordination and integration of signaling networks across different cell, tissue, and organ types within the plant. Bulk-cell and -tissue omics profiling, such as transcriptomics, proteomics, and metabolomics, have been used to uncover how gene products differentially accumulate across cell and tissue types. However, these bulk-level measurements only capture the average expression of a gene product within a cell population or tissue, masking the inherent heterogeneity of expression within single cells. Given that individual cells undergo developmental processes such as cell elongation and division at different rates, a single-cell expression profile is the logical next step toward obtaining a deeper understanding of the spatiotemporal response to various biological stimuli. The ability to quantify proteins from single cells has the potential to provide an important component of a Plant Cell Atlas (Rhee et al., 2019). Recent advances in protoplasting and sequencing technologies have allowed for single-cell transcriptomic studies in plants, but further developments are needed in the single-cell proteomics field to reach the same throughput as single-cell transcriptomic methods. One major issue for single-cell proteomics is that inherently low sample amounts (∼200 pg of protein per cell) challenge traditional sample preparation protocols and the sensitivity of current generation liquid chromatography–mass spectrometry (LC–MS) systems. Because of the challenges associated with measuring proteins from a single cell, groups have worked at the intermediate stage by isolating many cells of a single-cell type using laser capture microdissection or Fluorescent Activated Cell Sorting (FACS; Dai and Chen, 2012). Here, we provide an update on the state-of-the-art techniques in the single-cell proteomics field, discuss limitations for single-cell proteomics profiling in plant systems, and review potential applications for single-cell multi-omics profiling toward answering open questions in plant biology.

## Recent advances in low-input and single-cell proteomics

Multiple groups have made major improvements to the single-cell proteomics sample preparation and analysis protocols.

### Technological improvements facilitate direct single-cell proteome measurements

Several approaches have been developed for single-cell proteomics. One approach is to analyze the proteome of a single cell at a time using label-free quantification. However, label-free methods are inherently challenging for single-cell proteomics as the protein amount per cell is ∼200 pg for HeLa cells (Kulak et al., 2014) and ∼170 pg for *Arabidopsis thaliana* root protoplasts (8.40 ± 0.72 µg total protein extracted from 50,000 protoplasts with 5% sodium dodecyl sulfate and assayed by the Pierce BCA Protein Assay, *n *=* *3). Recent developments have trended toward reducing sample preparation volumes of 30 nL to 2 μL range to reduce sample loss (Li et al., 2018; Zhu et al., 2018b; Kelly, 2020; Brunner et al., 2021; Liang et al., 2021). It has also been found that using ultra-low LC flowrates (<100 nL min^−1^) and optimized gradients results in higher sensitivity (Zhu et al., 2018b; Kelly, 2020; Brunner et al., 2021; Liang et al., 2021).

On the MS side, gas-phase fractionation using ion mobility spectrometry (IMS) devices coupled to traditional LC–MS setups has proven to substantially increase ion selectivity and sensitivity by filtering out nonpeptide ions prior to MS analysis (Cong et al., 2020; Greguš et al., 2020; Brunner et al., 2021; Stejskal et al., 2021; Woo et al., 2021a). Using Nanodroplet Processing in One pot for Trace Sample (nanoPOTS) (see “Automated processing for small-volume samples”) sample preparation and optimized settings on a high field asymmetric IMS interface coupled to an Orbitrap Eclipse Tribrid MS identified over 1,000 protein groups from single HeLa and motor neuron cells (Cong et al., 2020). Likewise, a recent report used a modified trapped IMS TIMS-qTOF MS and a data-independent acquisition (DIA) parallel accumulation—serial fragmentation (diaPASEF) scheme (Meier et al., 2020) to quantify up to 1,400 protein groups per single cell (Brunner et al., 2021).

Acquiring MS data in DIA mode can reduce stochasticity in peptide identifications and can be more sensitive than typical data-dependent acquisition (DDA) for single-cell analysis (Saha-Shah et al., 2019). Although approaches for generating in silico spectrum libraries for DIA analyses exist (Demichev et al., 2020; Pino et al., 2020; Yang et al., 2020b; Zhang et al., 2020; Mehta et al., 2021), the development of cell type-specific DDA spectral libraries for DIA may reduce false positives and increase the certainty that particular proteins are expressed in specific cells (Rosenberger et al., 2017; Ge et al., 2020). These studies represent substantial advancements in single-cell analysis and further technological improvements to instrumentation and data analysis pipelines will increase the detection of low-abundance proteins from a single cell.

### Multiplexed single-cell proteomics with isobaric labeling

Multiple groups have developed single-cell proteomics methods that rely on sample multiplexing using isobaric labeling reagents such as Tandem Mass Tags (TMTs). Sample multiplexing enables greater throughput and a lower amount of LC–MS time per cell. While isobaric labeling enables multiplexing it is currently limited to 16 labels with up to 18 available in the near future (Li et al., 2021). Furthermore, multiplexing enables incorporation of an isobarically labeled carrier, often shortened to isobaric carrier, to boost signal in order to enhance peptide detection in MS scans. This carrier sample often consists of peptides from 25 to 200 cells, which are multiplexed with single-cell samples and processed in parallel. One of the first methods to implement an isobaric carrier for single-cell proteomics was Single-Cell ProtEomics by Mass Spectrometry (SCoPE-MS; Budnik et al., 2018; Slavov, 2021; Specht and Slavov, 2021; Specht et al., 2021). This approach has been used by several groups to answer different biological questions and serves as the building block for future isobaric carrier-based designs (Dou et al., 2019; Tan et al., 2019; Vitrinel et al., 2020; Yang et al., 2020a; Schoof et al., 2021). One note of caution is that while carriers enhance peptide identification, high carrier levels can negatively impact quantitative accuracy. This arises from Orbitraps having a fixed ion capacity. Thus, when high carrier amounts are used the ions from the single-cell samples are diluted out and not effectively detected. Indeed, several groups have worked to characterize optimal carrier amounts (Dou et al., 2019; Tsai et al., 2020; Cheung et al., 2021; Hartlmayr et al., 2021; Specht and Slavov, 2021). In particular, increasing the automatic gain control and ion injection time over settings typically used in bulk experiments improves quantitative accuracy. To aid in selecting optimal acquisition settings, which is instrument-dependent, several software packages have been developed including Single-Cell Proteomics Companion and Data-driven Optimization of MS (DO-MS; Huffman et al., 2019; Cheung et al., 2021).

**Figure 1:**
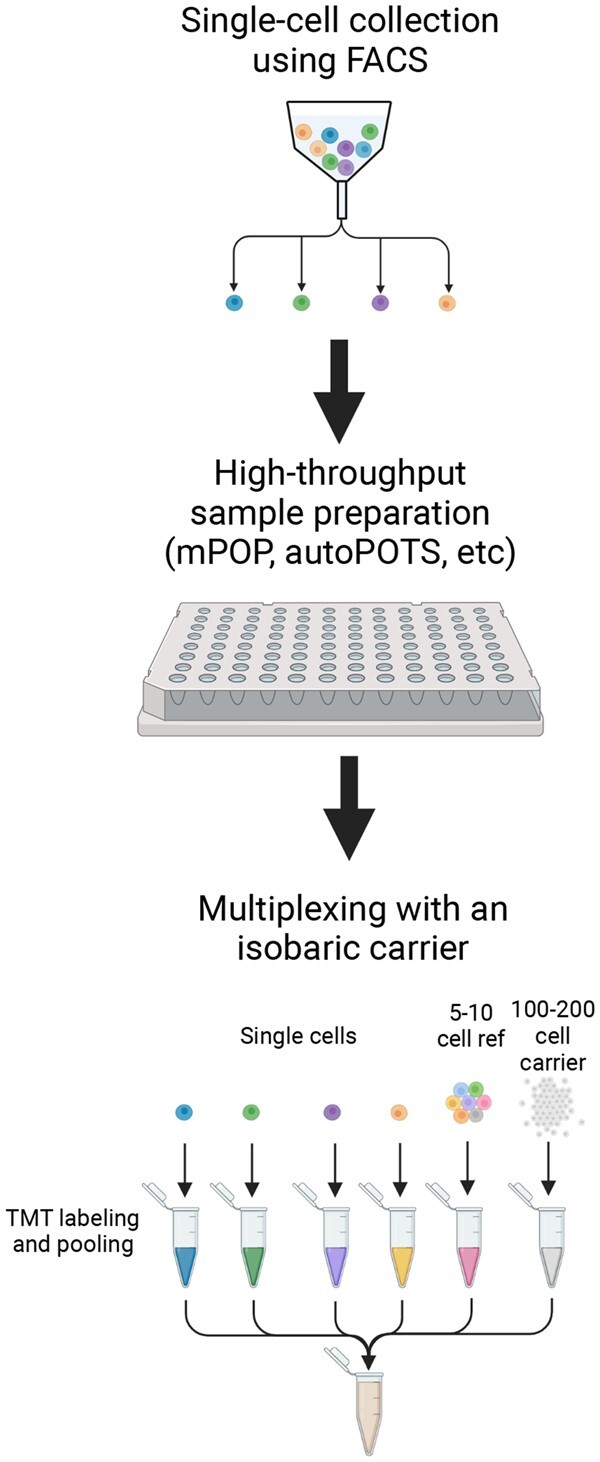
An example of single-cell proteomics sample preparation using multiplexing with an isobaric carrier. Single cells are collected using technology such as FACS and deposited into a 96- or 384-well plate for high-throughput sample preparation. During sample preparation, individual cells are lysed either manually or using automated protocols. Individual cells are then labeled using TMT isobaric labels. A reference sample of 5–10 cells and a carrier sample of 100–200 cells are run alongside the single cells to normalize total protein abundance between runs and increase throughput.

Recently, the group behind SCoPE-MS has made multiple improvements to increase the number of cells and proteins sampled while maintaining the affordable cost and the number of ion measurements for quantitative analysis of MS scans. In the original SCoPE-MS protocol, single mammalian cells were manually picked and lysed mechanically using sonication (Budnik et al., 2018). This new method, SCoPE2, improves on the reliability and reproducibility of single-cell collection, by directly depositing the single cells on 96- or 384-well plates after cell sorting, as well as incorporating the cell lysis method Minimal ProteOmic sample Preparation (mPOP) method (Specht et al., 2018, 2021; Figure 1). mPOP lyses cells using a freeze-heat cycle in water which removes the need for a cleanup step before MS analysis. Additionally, mPOP has the potential to be fully automated. The other main experimental optimization was introducing a reference channel across all of the TMT runs that is approximately five-fold more abundant than the single-cell samples (e.g. five cells in the reference sample versus one cell in the single-cell samples). This higher abundance improves the counting statistics, and therefore quantification, while remaining similar enough to single-cell profiles to not cause any major statistical issues (Figure 1).

SCoPE2 also implements some improvements for MS detection and identification. First, changes were made to the nanoflow-LC (nano-LC) gradient, peptide separation, and isolation window to measure more cells over time and improve ion isolation and quantification. Second, DO-MS was implemented to allow parameters to be individually tuned based on issues in the MS scans (Huffman et al., 2019). Finally, assignment of peptide sequences to MS spectra was improved using Data-driven Alignment of Retention Times for Identification, which uses Bayesian methodology to incorporate retention time and improve peptide-spectra matches (Chen et al., 2019a).

These improvements in SCoPE2 led to one of the largest, most comprehensive single-cell proteomics datasets to date which quantified over 3,000 proteins from almost 1,500 single cells in 10 d of instrument time (Specht et al., 2021). The benefits of multiplexing with isobaric carriers are further discussed in (Slavov, 2021). In general, TMT multiplexing with a carrier/booster channel has enabled several groups to analyze approximately 200 single cells per day with about 1,000 proteins measured per cell (Schoof et al., 2021; Singh, 2021; Specht et al., 2021).

### Automated processing for small-volume samples

One of the key components of SCoPE2, mPOP, allows for high-throughput processing of samples in volumes as low as 1 μL. Other groups have developed automated systems of sample preparation that improves on mPOP by using volumes in the nanoliter scale. While this small sample volume increases concentration and decreases losses due to surface contact, these methods often require custom-built robotics, pipettes, and plates. Thus, recent work has sought to improve the accessibility of these automated pipelines while maintaining the benefits of working in the nanoliter range.

nanoPOTS is one such automated sample processing technology developed for single-cell proteomics. nanoPOTS uses a custom-built nanoliter pipetting robot to deposit volumes <200 nL into a custom built nanochip, where each nanowell is surrounded by a hydrophobic barrier. Samples can then be processed using nanoLC-MS (Zhu et al., 2018a; Kelly, 2020). This technology has resulted in over 200 protein groups identified from single HeLa cells and over 1,500 groups from ∼10 HeLa cells (Zhu et al., 2018a). Additionally, nanoPOTS has been coupled with TMT isobaric labeling as in SCoPE-MS to measure protein expression on a high-throughput scale, quantifying over 1,200 proteins from over 60 single cells (Dou et al., 2019). While nanoPOTS improve detection of proteins from single cells, the custom hardware and delicate protocol have limited its adoption by other groups.

To improve the accessibility of nanoPOTS, its developers created Automated Processing in One pot for Trace Samples (autoPOTS), which uses a low-cost commercial robot and a commercial autosampler (Liang et al., 2021). The sample processing workflow is almost unchanged from nanoPOTS with the exception that volumes had to be upscaled to the low-microliter range to accommodate a commercial setup. Additionally, autoPOTS uses sealed-well plates to limit loss of sample via evaporation during incubation steps at elevated temperatures. autoPOTS performed comparably to nanoPOTS on samples composed of 1–500 HeLa cells with an ∼25% reduction in peptide coverage for single cells (Liang et al., 2021).

While methods like autoPOTS and mPOP allow for low-cost, automated processing of single-cell samples, they do not perform as well as techniques like nanoPOTS, which take advantage of nanoliter volumes. Improvements continue to be made to the nanoPOTS method, such as the development of a new N2 nanochip, which further reduces the volume and increases the number of single cells that can be processed on one chip (Woo et al., 2021b). Other nanoliter volume sample processing methods have been developed by other groups and are discussed in (Kelly, 2020). Finally, a commercial system termed cellenONE that combines single-cell isolation capabilities and picoliter dispensing has recently been released and holds promise for adoption by nonspecialized groups to perform low volume manipulation of single-cell samples (Hartlmayr et al., 2021).

### Improvements to nano-HPLC columns (μPAC)

After sample preparation, peptides extracted from single cells are usually processed using the combination of nanoflow high-performance LC (nano-HPLC) with electro-spray ionization and tandem MS/MS. A key part of this process is the chromatographic protein separation, which depends on the composition of the column. Furthermore, it has been shown that using nonporous C30 particles in place of typical C18 particles greatly increases the number of detected proteins from 100 to 1,000 cells (Kawashima and Ohara, 2018). Additionally, micropillar array-based nano-HPLC cartridges called μPAC columns have recently been commercialized, improving the accessibility of these nonporous columns for the broader scientific community. Compared to conventional C18 columns, μPAC columns yielded almost twice as many peptide identifications and protein groups on 10 ng of a HeLa cell digest. μPAC columns also had approximately two-fold higher peptide precursor-ion intensities and previously unobserved retention-time stability (Stadlmann et al., 2019). Other studies have shown similar performance of μPAC columns on low-input proteomics samples (Beeck et al., 2019). A new generation of μPAC columns designed specifically for low-input samples has recently released which facilitated the identification of nearly 1,500 protein groups from just 250 pg of sample (Stejskal et al., 2021). Thus, these nonporous chromatographic columns should be strongly considered for single-cell proteomics.

## Remaining challenges of single-cell omics in plant systems

While the recent improvements to single-cell proteomics sample preparation and analysis are promising, plant systems present unique challenges for single-omics experiments.

### Obtaining individual cells from plant tissue

**Figure 2:**
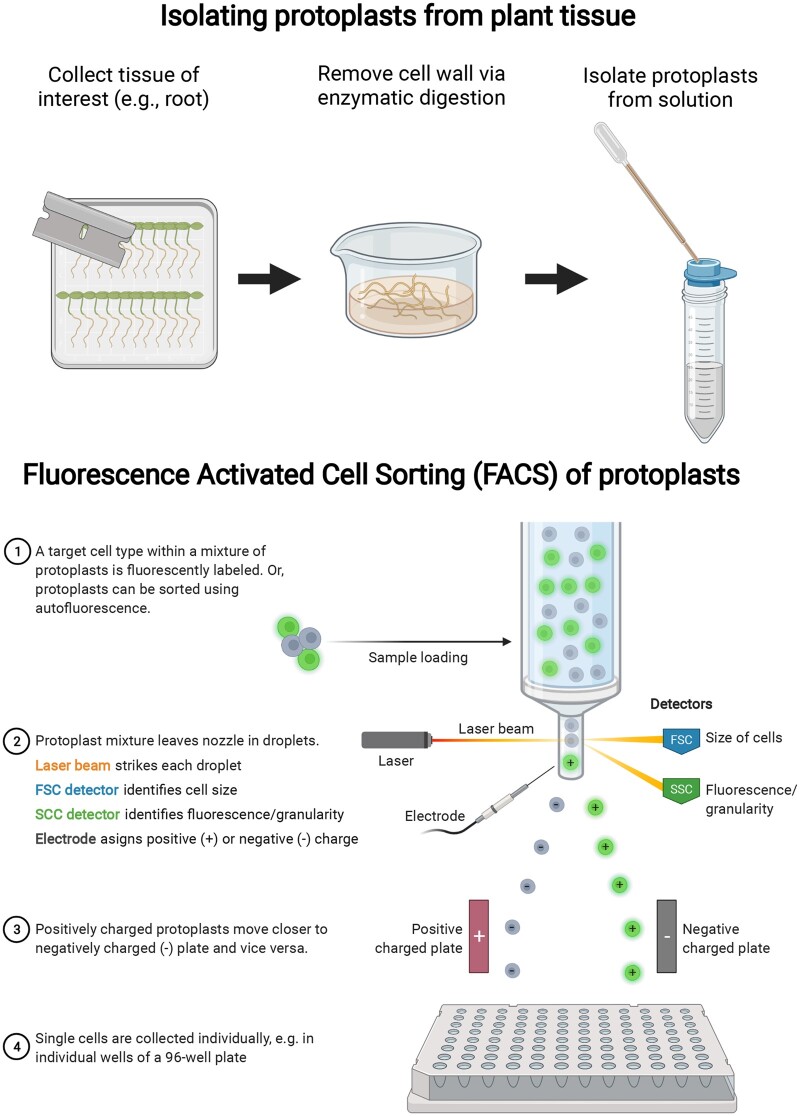
Protoplast isolation from plant tissue and FACS to isolate single plant cells. Plant tissue, such as root or leaf, is digested in an enzymatic solution to remove the cell walls. Protoplasts are then filtered through a cell strainer and collected for downstream analysis. During FACS, cells can be separated based on different features such as the presence of a fluorophore, cell size, cell shape, and/or charge. These parameters can be tuned to ensure that only live, individual cells are collected, removing contaminants such as clumps of cells or dead cells.

One of the most common methods for obtaining single cells in both animal and plant systems is FACS; Figure 2). During FACS, single cells are individually sorted into collection tubes or wells using either autofluorescence, when interested in all cell types, or a cell-specific or tissue-specific fluorescent marker when interested in a subset of cell types. Preparing cells for FACS from plant tissue introduces an additional challenge as plant cells contain cell walls. The cell wall must be removed from the membrane-encapsulated cell, called a protoplast, before the cells can be sorted using FACS or other methods (Figure 2). Protoplast extraction methods coupled with FACS have been developed for numerous plant organisms such as Arabidopsis (Birnbaum et al., 2005; Yoo et al., 2007) and maize (*Zea mays*; Ortiz‐Ramírez et al., 2018), but these methods have not been widely adopted across different plant species. Additionally, these methods are time-consuming, as the cell walls must be slowly dissolved using enzymes to avoid damaging the protoplasts. Once isolated, protoplasts are extremely sensitive and must be processed in a tight time window to avoid environmental and mechanical stress that could affect downstream measurements. It has been shown that the protoplasting process can result in approximately 300–400 altered transcripts but did not majorly impact downstream analysis and biological conclusions (Birnbaum et al., 2005; Yadav et al., 2009; Villarino et al., 2016). Furthermore, by comparing samples that have been protoplasted in the same manner, these technical effects are controlled for and should not majorly influence biological conclusion. While current protoplast isolation methods have been sufficient to build single-cell and single-nuclei RNA-seq atlases in Arabidopsis (Denyer et al., 2019; Zhang et al., 2019; Farmer et al., 2021; Lopez-Anido et al., 2021), maize (Satterlee et al., 2020; Marand et al., 2021; Xu et al., 2021), rice (*Oryza sativa*; Liu et al., 2021), and tomato (*Solanum lycopersicum*; Tian et al., 2020), optimization is necessary to isolate protoplasts from a range of species and tissues. Single-cell isolation methods from plants are further discussed in (Libault et al., 2017) and (Gurazada et al., 2021).

### Determining and detecting cell type-specific marker genes

**Figure 3:**
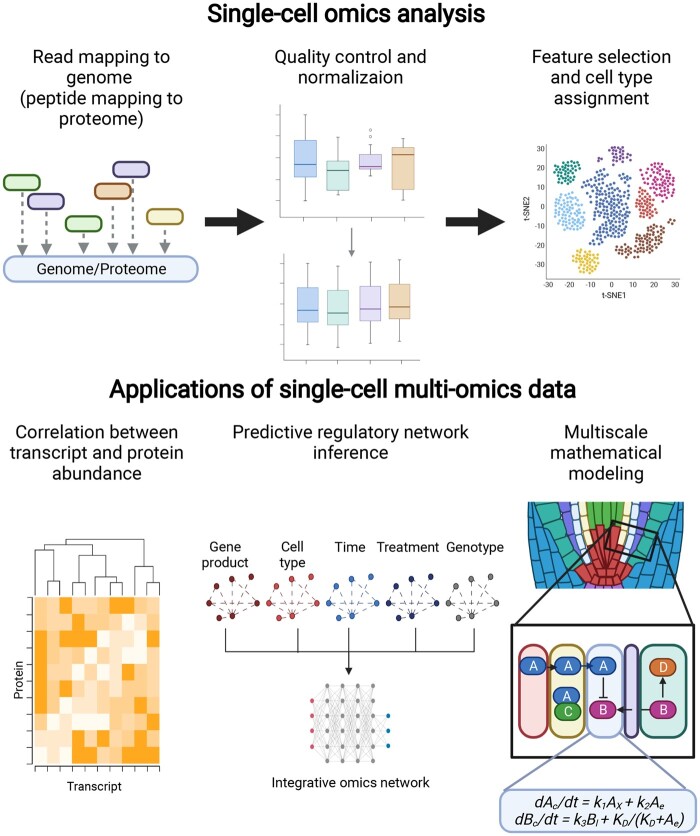
Analysis and applications of single-cell omics in plants. Single-cell transcriptomics and proteomics must be mapped to the reference genome (or proteome) and normalized before downstream analysis. Initial analysis of single-cell omics data includes selection of HVGs and cell-type assignment. Further applications of single-cell omics data include, but are not limited to, correlation analyses, predictive network inference, and multiscale mathematical modeling such as ABMs.

An important component of any single-cell omics analysis is the ability to assign individual cells to their specific cell type so that their gene expression may be compared to cells within the same and between different populations. This is usually performed using a selection of 1,000–5,000 highly variable genes (HVGs) that are truly informative of the variability of the data. Only these HVGs are used for the downstream analysis, including visualization, cluster analysis, and trajectory inference (Figure 3). Once the cells are grouped based on the expression of HVGs, the identity of each cluster can be inferred using the expression of known cell type-specific marker genes. However, there are limitations to this process, especially regarding plant single-cell omics data. First, the desired marker genes must be in the set of HVGs that are consistently measured across individual cells. This is not always the case as single-cell omics data tends to suffer from large dropout effects (large numbers of zero values; Brennecke et al., 2013; Qiu, 2020). Second, the marker genes must be consistently expressed in the cell type of interest, which can be difficult due to the higher amount of noise and stochasticity in expression measurements versus cell type-specific RNA-seq (Brennecke et al., 2013). Finally, there must be a curated list of marker genes for the specific cell types and organism of interest. This is a major issue in plant biology: while some organisms and cell types have a breadth of marker genes, such as the Arabidopsis root, many others do not. Additionally, most of the cell type-specific profiling in plants has been at the transcript level (Libault et al., 2017). There currently are not as many markers for other levels of single-cell omics data, such as proteomics. Thus, as these single-cell proteomics methods are developed and used in plant systems, they will likely need to be coupled with cell type-specific profiling to identify the necessary markers for single-cell classification. Single-cell transcriptomics in plants is further discussed in (Luecken and Theis, 2019).

### Accessibility and availability of single-cell data analysis methods

A final consideration for single-cell omics is the choice of methods for downstream statistical analysis. The single-cell RNA-seq community has developed a plethora of computational tools for the various steps of the data processing and analysis process, including read mapping, drop-out/missing value imputation, normalization, and differential expression testing (Figure 3). As of this writing, the scRNA tools database (https://www.scrna-tools.org/) has documented over 900 different single-cell RNA-seq analysis methods, 51% of which have been published in a peer-reviewed journal. These methods have a wide range of availability (open-access versus commercially licensed) and are programmed in a number of different languages (R and Python are the two most common; Zappia et al., 2018). Furthermore, different combinations of the available methods can result in significantly different cell classifications and numbers of differentially expressed genes, greatly affecting the biological interpretation of the results (for more guidance on choice of single-cell statistical methods, please see Vieth et al., 2019). Currently, experimentalists must combine all of these individual components into one functional workflow, which can be challenging without a strong bioinformatics background. Additionally, all of the aforementioned methods have been developed for single-cell transcriptomics. Other single-cell omics data, such as proteomics, will not necessarily follow the same statistical distributions and have the same statistical assumptions as transcriptomics data. Furthermore, gene expression control tends to result in large fold-changes in transcript levels but relatively smaller changes in protein amounts (Vitrinel et al., 2019). As a result, integration of data types often requires appropriate scaling for joint analyses. Thus, these methods must be carefully validated on other types of single-cell omics data before they are used for large-scale, cell-specific, multi-omics analysis in plants.

## Applications of single-cell proteomics in plant biology

The combination of single-cell transcriptomics and proteomics data can be used to answer a breadth of fundamental questions in plant biology.

### Correlation between transcript and protein abundance at the single-cell level

First, these data will reveal the correlation, or lack of, between transcript and protein abundance at the single-cell level (Figure 3). Similar to studies in nonplant systems, there has been substantial work in plants which shows that transcript and protein abundance only moderately correlate across the different organs and tissues of the plant (Petricka et al., 2012; Ponnala et al., 2014; Walley et al., 2016; Seaton et al., 2018; Mergner et al., 2020; Zander et al., 2020). One of the most thorough analyses of transcript and protein abundance was performed in maize, where the transcriptome, proteome, and phosphoproteome of 23 different tissues were profiled. It was shown that most tissues have only a low to moderate correlation (Pearson coefficient of 0.40–0.60) between transcript and protein abundance. Further, it was shown that networks inferred using transcript and protein abundance have greatly different topologies, sharing only 11% of inferred edges, and that a network integrating these different omics data had higher precision and recall than the individual omics networks alone (Walley et al., 2016). Similar results were shown in an integrative omics profile of jasmonic acid signaling in Arabidopsis, where the networks inferred using transcriptomic, proteomic, and phosphoproteomic data had limited shared edges (Zander et al., 2020). Other groups have examined the relationship between the transcriptome and proteome and have found similar magnitudes of the Pearson coefficient in the Arabidopsis root (0.19–0.36; Petricka et al., 2012), across different Arabidopsis tissues (0.28–0.7; Mergner et al., 2020), between different photoperiods in Arabidopsis rosettes (0.47–0.86; Seaton et al., 2018), developing maize leaf (0.45–0.65; Ponnala et al., 2014), maize root and embryo (0.35 and 0.43, respectively; Jia et al., 2018), and across a maize diversity panel (0.13; Jiang et al., 2019). Recent analyses of single-cell transcriptomics and single-cell proteomics in animal systems show that while some proteins and transcripts correlate well, others do not (Specht et al., 2021). In addition, technical differences in how transcripts and proteins are measured could contribute to the lack of correlation specifically at the single-cell level (Brunner et al., 2021). Thus, based on these bulk-tissue studies, we are likely missing a crucial piece of biological signaling and regulation in plants by measuring only the transcriptome.

In Arabidopsis, there are a number of biological processes that are controlled through transcription factors (TFs) whose proteins are cell-to-cell mobile. One of the best examples of this is SHORTROOT (SHR), a mobile TF that controls cell patterning and division in the Arabidopsis root stem cell niche and ground tissue (Gallagher et al., 2004; Levesque et al., 2006; Clark et al., 2016; Clark et al., 2020a). Another example is the TFs CAPRICE (CPC) and GLABRA3 (GL3), which control root hair patterning (Kurata et al., 2005; Savage et al., 2008). Crucially, these TFs have substantially different transcript and protein distributions. For example, SHR transcript is localized only to the vasculature, whereas its protein can move to all of the surrounding cell types. By profiling both transcriptomics and proteomics at the single-cell level, we may identify additional proteins like SHR, CPC, and GL3, which move between different cell types to control cell-specific regulatory networks.

### Integration of different single-cell omics measurements in predictive networks

As shown by (Walley et al., 2016), the integration of different omics-level measurements is crucial to deepen our understanding of biological processes within the plant. However, most of the regulatory networks and mathematical models that have been constructed to describe biological processes are built entirely on transcript abundance data. Thus, recent work has sought to integrate transcriptomics and proteomics data into one comprehensive regulatory network (Figure 3). Specifically, we developed a computational method named Spatiotemporal Clustering and Inference of Omics Networks (SC-IONs), which allows one to construct integrative omics networks from bulk-tissue transcriptome and proteome data (Clark et al., 2020b). Additionally, integrative Dynamic Regulatory Events Miner combines static protein–DNA interaction data with time series expression data including transcriptomics, proteomics, epigenomics, and/or single-cell RNA-Seq to generate dynamic regulatory networks (Ding et al., 2018). Methods such as these could be adapted for single-cell integrative omics data and could potentially also incorporate single-cell cis-regulatory information generated with scATAC-Seq (Marand et al., 2021; Marand and Schmitz, 2022).

While these integrative omics networks are informative and allow us to identify causal genes, they are still limited by the scope of the data, which tend to only contain three to four biological replicates per treatment group. On the other hand, single-cell measurements can be treated as individual biological replicates, meaning that one can now infer a network from thousands of individual cells. This increase in data results in networks with higher precision, recall, and predictive power. Numerous network inference methods have been developed for single-cell RNA-seq data, such as SCENIC (Aibar et al., 2017) and SCODE (Matsumoto et al., 2017), which could be modified for multi-omics single-cell data in a similar fashion as SC-ION. However, important considerations must be made given that single-cell data are inherently more stochastic than bulk-cell data. Single-cell transcriptomics network inference methods are further discussed in (Chen et al., 2019b).

### Multiscale mathematical modeling at the single-cell resolution

Many mathematical models have been developed for various biological processes in plants at the cell-specific, tissue-specific, and organ-specific levels. These quantitative models cover a range of processes including root stem cell division (Clark et al., 2016, 2020a), root hair patterning (Savage et al., 2008), and hormone gradients within the root such as auxin (Grieneisen et al., 2007; Mironova et al., 2010; Clark et al., 2014) and gibberellin (Rizza et al., 2021). While these models have been used to make predictions about the cell-specific nature of these biological processes, they are limited by the mostly tissue- or cell type-specific, transcript-level data used to construct and validate the models. The incorporation of single-cell proteomics data would improve the predictive power of these mathematical models and lead to a deeper understanding of how these biological processes vary in a cell-specific manner.

One type of mathematical model that lends itself particularly well to single-cell omics data is the agent-based model (ABM). In an ABM, the individual cells of an organism are treated as the agents, and each cell has a set of equations (usually Ordinary Differential Equations), which represent the individual regulatory networks within each cell (Figure 3). There are also equations connecting different cells, representing processes like mobile signals and proteins, and developmental processes such as cell division and growth can be incorporated into the model. Recently, an ABM was developed for Arabidopsis root stem cell division (Van den Broeck et al., 2021). While this ABM uses cell type-specific transcriptomic data, it illustrates the potential for modeling at the single-cell, multi-omics level in plant systems. However, just as with network inference methods, these models will need to be adapted to deal with the inherent stochasticity of single-cell data.

## Future developments and recommendations

Based on the current state-of-the-art of single-cell proteomics, we foresee the following future developments and make recommendations on how single-cell proteomics can be incorporated into the plant biology field.

### Single-cell measurements of post-translational modifications

While these recent advances in single-cell proteomics are promising for the plant biology community, they are unable to capture the effect of post-translational modifications (PTMs), such as phosphorylation, ubiquitination, and acetylation, on plant signaling mechanisms. These PTMs have been shown to be important for a variety of biological processes in plants, including plant immunity (Dodds and Rathjen, 2010; Sato et al., 2010; Tsuda and Katagiri, 2010; Song and Walley, 2016; Walley et al., 2018) and hormone signaling (Tal et al., 2020; Song et al., 2021). However, single-cell analysis of PTMs continues to be limited by the large amount of starting material required for PTM enrichment relative to total protein (Low et al., 2021). Thus, innovative sample preparation protocols are needed to detect PTMs from single cells.

### Increased accessibility of nanoliter-scale methods

Single-cell sample preparation at the nanoliter-scale has been a major contributor to the rise of single-cell proteomics (see above). However, these nanoliter-scale methods require custom equipment and technology that is not widely accessible. While there are protocols that use microliter volumes, they do not perform as optimally as nanoliter-scale methods (Liang et al., 2021). Adapting nanoliter-scale protocols to use more accessible equipment and materials would help to expedite the adoption of single-cell proteomics in more labs.

### Further development of mass spectrometry imaging

Mass Spectrometry Imaging (MSI) is another approach to analyze a range of types of molecules including proteins, peptides, glycans, lipids, etc. with high spatial resolution (Dilillo et al., 2017; Keller et al., 2018; Taylor et al., 2021). There are a number of MSI techniques that enable in situ analysis of molecules of interest using chemical desorption and/or ionization to introduce analytes into the MS. An advantage of MSI is that chemical desorption and/or ionization is carried out on intact tissue or tissue sections, which eliminates the need to enzymatically digest cell walls. While MSI approaches are being developed for protein measurements they currently provide limited coverage of only a handful of proteins. Thus, further refinement of MSI is necessary for high-throughput measurements of single-cell proteomes.

### A unified and integrated single-cell data analysis platform

One of the major hurdles in single-cell omics data analysis is the large number of available methods. Although databases like scRNA-tools exist, it is still assumed that experimentalists can assemble a single-cell analysis workflow on their own. Without a strong bioinformatics and statistics background, this could result in groups using inappropriate methods for their specific experimental setup and data distribution, which could alter the biological conclusions. Further, experimentalists may not even be able to use their preferred analysis methods due to lack of accessibility (i.e. not open access or in an unfamiliar computational language). Recent work has standardized the analysis of single-cell proteomics data collected using the SCoPE2 method into an R package called scp (Vanderaa and Gatto, 2021). However, this is only one solution for a specific methodology that still relies on the background computational knowledge of the experimentalist. We propose that the single-cell omics community creates an integrated single-cell data analysis platform that is widely accessible to scientists from all fields. Additionally, future work should adapt these methods to make them suitable for analysis of single-cell proteomics data.

### Stochastic modeling of single-cell omics expression

One application of single-cell omics data is its incorporation into network inference and mathematical modeling across different areas of plant biology. Importantly, single-cell data differ from bulk-cell data due to their inherent stochasticity. Most of the network inference methods and models that have been developed for bulk-cell data assume that the individual biological replicates do not suffer from this large amount of noise. Thus, using these methods directly on single-cell omics data is likely not statistically sound and could result in incorrect biological conclusions. We recommend that future work on network inference and modeling of single-cell omics data incorporates concepts from the stochastic modeling field in order to adequately account for the high amount of noise in single-cell data. Stochastic modeling of single-cell data is further discussed in (Hsu and Moses, 2021).

## Concluding remarks

Recent developments in the single-cell proteomics field put us one step closer toward its implementation in plants. However, there are still remaining challenges towards adapting single-cell proteomics methods in both plant and animal systems. There are also additional technical considerations given the different composition and sensitivity of plant protoplasts. Finally, the integration of these single-cell multi-omics data will require techniques from the field of systems biology, including bioinformatics, statistics, and mathematical modeling (see Outstanding Questions). Despite these challenges, single-cell proteomics profiling in plants is an exciting prospect that will allow us to answer many fundamental questions on a deeper spatiotemporal scale.

**Advances Box**

- Single-cell proteomics methods enabled quantification of over 3,000 protein groups per experiment at around 1,000 proteins per single cell in animal systems.
- Improvements in instrumentation and nanoliter sample volume processing have greatly increased the number of proteins detected at the single-cell level.
- There has been an increase in the number of single-cell transcriptomic atlases generated in plants.
- Many computational methods have been developed for the statistical analysis of single-cell transcriptomics.
- The combination of single-cell transcriptomics and proteomics can answer fundamental questions in plant biology.

**Outstanding Questions**

Our review raises the following questions for the single-cell omics and plant biology communities. How can we:

- Make nanoliter-scale sample preparation methods more accessible?
- Adapt single-cell proteomics methods for plant material?
- Improve single-cell isolation protocols in plants, especially in nonmodel plant species?
- Identify more cell type-specific marker genes in plants at both the transcript and protein abundance level?
- Accommodate the inherent stochasticity of single-cell omics data in the downstream statistical analysis?
- Create a unified, easy-to-use data-processing workflow for single-cell omics analysis?
- Make the needed technical advances to measure post-translational modifications on the single-cell level?
- Incorporate single-cell data into existing network inference methods and mathematical models?

## Notes

N.M.C. and J.M.E. made the figures. N.M.C., J.M.E., and J.W.W. wrote the manuscript.

The author responsible for distribution of materials integral to the findings presented in this article in accordance with the policy described in the Instructions for Authors (https://academic.oup.com/plphys/pages/general-instructions) is Justin W. Walley (jwalley@iastate.edu).
